# Intraoperative Indocyanine Green Imaging for the Evaluation of Blood Perfusion Area in Cancer of the Splenic Flexure With an Occluded Inferior Mesenteric Artery: A Report of Two Cases

**DOI:** 10.7759/cureus.30943

**Published:** 2022-10-31

**Authors:** Ryo Takahashi, Nobuji Kouno, Takumi Furuya, Takahisa Fujikawa

**Affiliations:** 1 Department of Surgery, Kokura Memorial Hospital, Kitakyushu, Fukuoka, JPN

**Keywords:** accessory middle colic artery, arteriosclerosis, endovascular aortic repair, post-operative bowel ischemia, indocyanine green fluorescence, inferior mesenteric artery occlusion, splenic flexure cancer

## Abstract

Radical resection for cancer of the splenic flexure requires careful consideration of the dissection line so that blood flow in the remnant bowel is maintained, particularly when the root of the inferior mesenteric artery (IMA) is already occluded. Intraoperative indocyanine green (ICG) imaging is a promising method for evaluating blood perfusion of organs and vessels. However, there are few reports on the use of ICG to determine the dissection line in patients with altered blood flow. In this article, we describe two cases of successful resection of splenic flexure cancer (SFC) in patients with an occluded IMA under ICG guidance.

Case one was a 76-year-old man with a diagnosis of stage III SFC who had previously undergone endovascular aortic repair without reimplantation of the IMA. Intraoperative ICG imaging revealed that the left side of the colon was perfused mainly by the left branch of the middle colic artery (MCA). We performed a hemicolectomy with preservation of the MCA-left colic artery (LCA) arcade and resected an enlarged lymph node *en bloc*. Case two was a 77-year-old man with a diagnosis of stage II SFC in whom the root of the IMA appeared to be occluded by arteriosclerosis. Computed tomography showed that the LCA was anastomosed to the accessory middle colic artery (AMCA) while the left branch of the MCA was joined to the marginal artery. Intraoperative ICG imaging revealed that the left side of the colon was perfused by the AMCA and not the MCA. By preserving the AMCA-LCA arcade, we were able to safely divide the left branch of the MCA. Both patients were discharged with no symptoms of bowel ischemia or recurrence of cancer during follow-up.

Interindividual variation in vessel branching patterns and dominant vessels in the descending and distal transverse colon may result from congenital factors or acquired disease. Detailed information on blood perfusion is required to avoid postoperative bowel ischemia. This report is the first to focus on patients with SFC and altered blood flow. We show that ICG imaging might be a reasonable option for determining an adequate surgical dissection area.

## Introduction

In radical surgery for colorectal cancer (CRC), the feeder vessels are commonly divided at the root, and perfusion of the remnant bowel is preserved by other vessels. However, some patients have an altered blood flow pattern and perfusion area because of cardiovascular disease or previous resection of CRC. Moreover, the typical division of vessels may cause postoperative bowel ischemia (BI), particularly in patients with splenic flexure cancer (SFC) and an occluded inferior mesenteric artery (IMA). Therefore, surgeons need to consider whether or where they should divide vessels.

Intraoperative indocyanine green (ICG) fluorescence imaging is emerging as an alternative to computed tomography (CT) angiography for the assessment of blood flow and branching patterns in arteries [1-3]. ICG imaging enables the identification of the vessels/area that should be preserved or dissected [4]. However, while there is substantial literature on the assessment of anastomotic perfusion to reduce leakage [5-7], there are few reports on the use of ICG imaging to determine the dissection line in patients with altered blood flow. Here, we report two cases of successful resection of the SFC in patients with an occluded IMA under intraoperative ICG guidance.

## Case presentation

Case one

**Figure 1:**
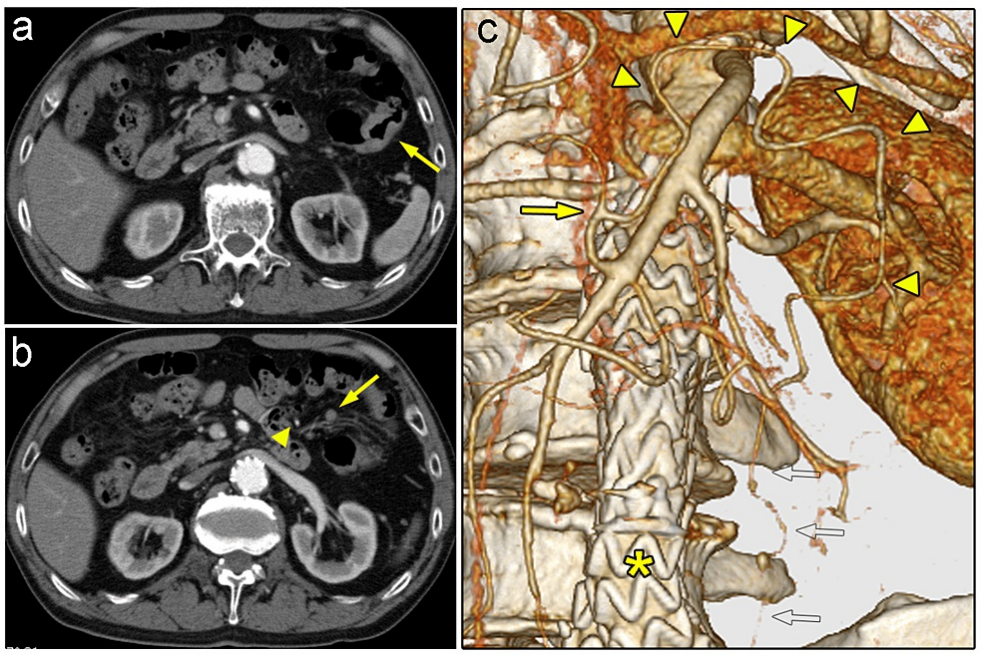
Preoperative images for case one a) A CT scan showing thickening of the wall at the splenic flexure (arrow). b) An enlarged regional lymph node (arrow) was detected between the tumor and the arterial arcade (arrowhead). c) CT angiography demonstrated that the left branch of the MCA (arrow) was anastomosed to the LCA via the arterial arcade (arrowheads). There was no sign of blood flow at the root of the IMA, and the SRA (open arrows) was barely patent because of aortic stenting (asterisk). CT: computed tomography; IMA: inferior mesenteric artery; LCA: left colic artery; MCA: middle colic artery; SRA: superior rectal artery

The patient was a 76-year-old man who visited our institution with a complaint of bloody stool. The patient was receiving clopidogrel as anti-platelet therapy for the previous lacunar infarction, and he had stage G3b chronic renal failure. Detailed examination revealed a neoplastic lesion involving almost the entire circumference of the splenic flexure (Figure 1a), and swelling of a regional lymph node (LN) (Figure 1b). The diagnosis was clinical T3N1aM0, stage IIIb SFC. The patient also had a history of endovascular aortic repair (EVAR) for abdominal aortic aneurysm (AAA) and therefore lacked antegrade blood flow at the root of the IMA. CT angiography showed that the left branch of the middle colic artery (MCA) was anastomosed to the left colic artery (LCA) and that the marginal artery was not dilated (Figure 1c). We expected that the left side of the colon would be perfused mainly by this MCA-LCA arcade.

**Figure 2:**
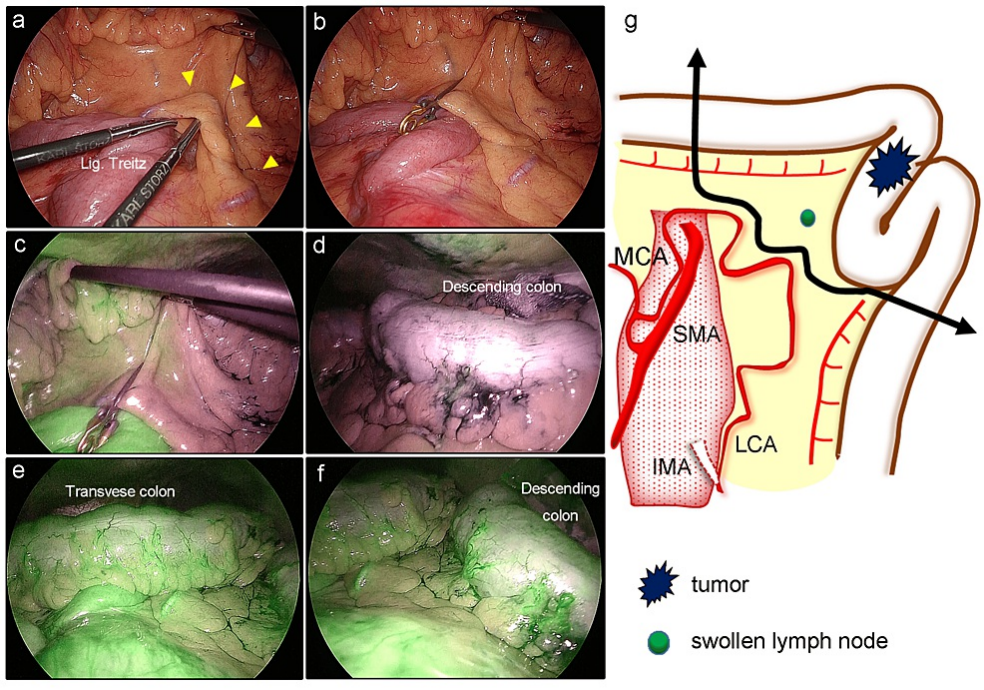
Intraoperative findings for case one a) The arterial arcade consisting of the MCA and LCA (arrowheads) was identified. b) The arcade was clamped to evaluate blood perfusion by ICG imaging. c) Thereafter, a clear demarcation was observed at the splenic flexure. d) The fluorescence intensity in the descending colon and sigmoid colon was markedly reduced. e, f) After the removal of the clamp, blood perfusion returned in the entire left side of the colon. g) The scheme of surgery. A colectomy was performed with dissection of a swelling LN en bloc while preserving the MCA-LCA arcade (double arrow). ICG: indocyanine green; IMA: inferior mesenteric artery; LCA: left colic artery; Lig.Treitz: Ligament of Treitz; LN: lymph node; MCA: middle colic artery; SMA: superior mesenteric artery

During the surgery, the left branch of the MCA was clamped using a laparoscopic approach (Figures 2a, 2b). On ICG imaging, there was a significant reduction in fluorescence intensity from the splenic flexure to the sigmoid colon (Figures 2c, 2d). After the removal of the clamp, perfusion of the entire left side of the colon was fully recovered (Figures 2e, 2f). Considering the balance between the patient’s general condition and the surgical invasiveness, we performed a colectomy with dissection of a swelling LN en bloc with preservation of the MCA-LCA arcade, instead of resection of the entire left-side colon (Figure 2g).

The postoperative course was uneventful. Pathological examination of 15 harvested LNs revealed that one was metastatic; this LN corresponded to the enlarged LN seen on preoperative CT. The patient did not receive any adjuvant chemotherapy because of chronic renal dysfunction but has shown no signs of recurrence during 15 months of follow-up.

Case two

**Figure 3:**
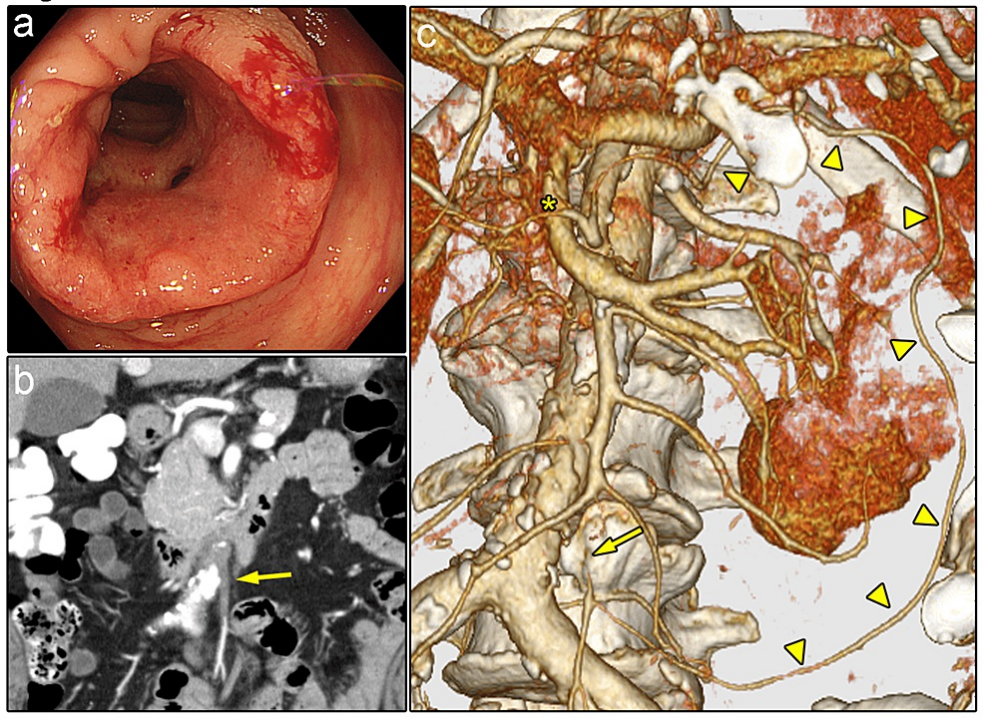
Preoperative images for case two a) Colonoscopy revealed the growth of an advanced tumor. b) CT showed that the root of the IMA was occluded (arrow). The SRA was patent. c) CT angiography demonstrated no blood flow at the root of the IMA (arrow). The distal branches were compensated. The LCA formed an arterial arcade with the AMCA (arrowheads), which shared the same origin as the MCA in this case. The asterisk indicates the left and right branches of the middle colic artery. AMCA: accessory middle colic artery; CT: computed tomography; IMA: inferior mesenteric artery; LCA: left colic artery; MCA: middle colic artery; SRA: superior rectal artery

The case was a 77-year-old man who was admitted to our institution for investigation of symptoms of angina and found to have anemia during his hospital stay. The patient was previously diagnosed with moderate aortic regurgitation and received clopidogrel. Detailed examination revealed advanced colon cancer on the splenic flexure (Figure 3a). CT did not show any signs of regional LN swelling or distant metastasis but revealed that the root of the IMA was occluded, probably by thrombus or arteriosclerosis (Figure 3b). The LCA and the superior rectal artery were patent and the LCA was anastomosed to the accessory middle colic artery (AMCA), the origin of which was shared with the MCA while the left branch of the MCA joined the marginal artery of the transverse colon (Figure 3c).

**Figure 4:**
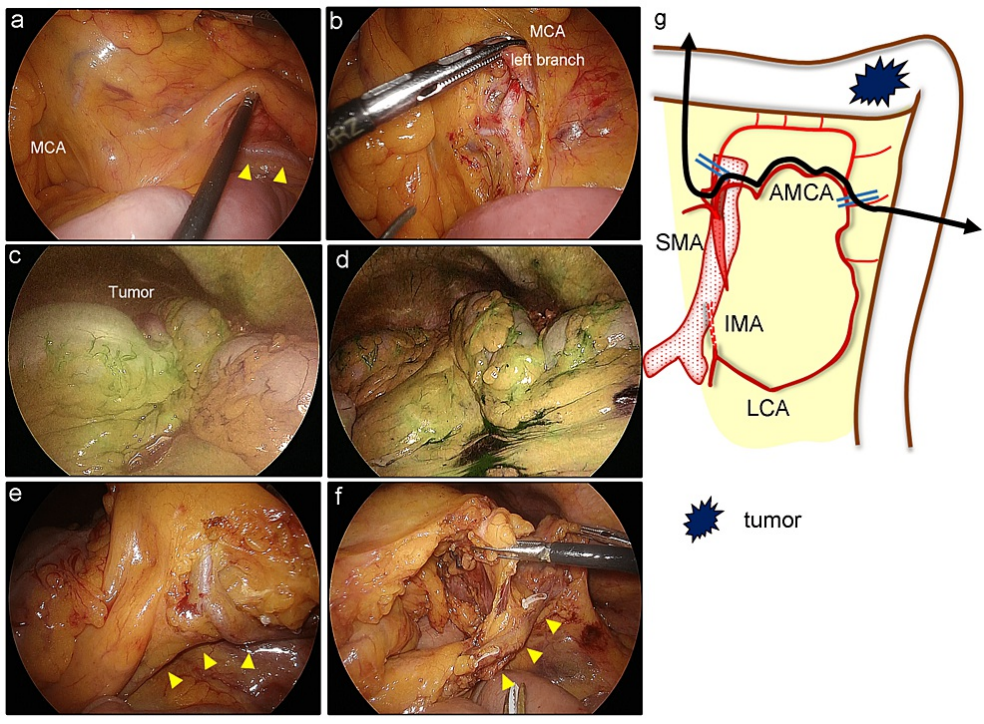
Intraoperative findings for case two a) The MCA and AMCA (arrowheads) were separately identified. b) The left branch of the MCA was further isolated for clamping. c) The left branch of the MCA and AMCA was clamped and ICG was administered. Fluorescence intensity was significantly reduced in the descending colon and sigmoid colon. d) After the removal of the clamp, blood perfusion returned in the entire left side of the colon. e) The marginal artery that branched from the arterial arcade (arrowheads) was identified and divided. f) The mesentery was dissected along the distal side of the arterial arcade (arrowheads). g) The scheme of surgery. The arteries were clamped at the points which were indicated by double bars. The left branch of the MCA and the marginal artery were divided, and the AMCA-LCA arcade was preserved (double arrow). AMCA: accessory middle colic artery; ICG: indocyanine green; IMA: inferior mesenteric artery; LCA: left colic artery; MCA: middle colic artery; SMA: superior mesenteric artery

Surgery was performed using a laparoscopic approach. The AMCA and the left branch of the MCA were identified (Figures 4a, 4b). When the left branch of the MCA and the AMCA was clamped and intravenous ICG was administered, we observed a significant reduction of fluorescence in the descending colon and sigmoid colon (Figure 4c). We then declamped the AMCA alone and confirmed that bowel perfusion was fully recovered (Figure 4d). Based on these findings, we concluded that the left side of the colon was not perfused by the MCA. To avoid excessive surgical invasiveness, we divided the left branch of the MCA and the marginal artery while preserving the AMCA-LCA arcade (Figures 4e, 4f, 4g).

Paralytic ileus (Clavien-Dindo grade 2) occurred on postoperative day three but improved rapidly. The patient was discharged on postoperative day 10. Pathological examination of 16 harvested LNs revealed no metastasis. There have been no signs of recurrence during six months of follow-up.

## Discussion

Blood flow in the descending colon and distal transverse colon is supplied mainly by arterial branches from both the superior mesenteric artery (SMA) and IMA and partially by the internal iliac artery (IIA). However, arterial communications, including the collateral arteries (e.g., Riolan’s arch) and marginal vessels (e.g., the artery of Drummond) and their branching patterns or dominant vessels in each area, vary among individuals [8]. In addition to these congenital factors, some elderly patients have acquired cardiovascular disorders such as arteriosclerosis or AAA. In a study by Zhang et al., angiography revealed that 25 of 154 elderly patients had arteriosclerotic lesions in the IMA, including 10 stenoses (6.5%), three occlusions (1.9%), and 12 plaques (7.8%) [8]. EVAR has been widely performed in patients with AAA in addition to traditional open repair. A recent study found that 78.5% of infrarenal AAAs were treated by EVAR [9]. When endovascular treatment is performed, antegrade flow in the IMA is sacrificed without reimplantation. Prior CRC resection is another cause of the lack of flow in the IMA. In patients with an occluded IMA, surgeons should carefully consider postoperative perfusion of the remnant bowel, especially on the anal side. Sakurazawa et al. investigated blood flow in the rectal wall during AAA repair and found that the SMA was the main supplier and provided adequate blood flow in the rectum in 81% (67/83) of cases [10]. The IIA is another candidate for blood supply in the descending colon. Ohno et al. reported a case of post-EVAR SFC resection using preoperative CT angiography. They observed arterial blood flow up to 30 cm from the peritoneal reflection by pulsatile bleeding at the cutting edge of the marginal artery [1]. More recently, this issue was further investigated utilizing ICG fluorescence imaging, which has been developed for the investigation of anastomotic perfusion, especially in left-sided colorectal surgery. Munechika et al. performed a prospective pilot study on the cancer of the descending colon. After dividing the blood perfusion from SMA, IMA was temporarily clamped and the blood flow from IIA was assessed by ICG. They demonstrated that the ICG fluorescence reached 17-66 cm from the peritoneal reflection and IMA high ligation was safely performed in all 20 cases without anastomotic leakage [11]. However, as our cases and some literature indicate, IIA does not necessarily provide a reliable blood supply for the entire descending colon [12].

The AMCA is typically identified as the artery that arises separately from the SMA near the first jejunal artery proximal to the MCA and runs along the inferior border of the pancreas toward the splenic flexure [2,13]. A systematic review by Cheruiyot et al. found that AMCA was present in approximately 25% of cases [13]. When present, the AMCA might serve as an alternative source of collateral blood flow between the SMA and IMA; therefore, in some cases, the MCA and part of the marginal artery could be divided.

Altered blood perfusion must also be considered in patients undergoing vascular surgery for AAA after a prior left-sided colectomy. The absence of flow in the SMA is a well-recognized contraindication of EVAR. Brewster et al. warned in the early 1990s that prior colon resection could impair collateral circulation [14]. Since then, however, most large-scale studies of the risk factors for BI after AAA repair has not examined the impact of prior colectomy [9,15-17], possibly because the low incidence of post-EVAR BI (0.6-2.1%) [9,15-18] precludes detailed analysis of risk factors. Becquemin et al. found no significant increase in BI in their series of patients with prior colectomy but considered that the impact of collateral ligation between the SMA and IMA/IIA by prior colectomy has been underestimated [18]. Several reports have mentioned the possibility of a correlation between post-EVAR BI and previous colectomy [18-20]. Indeed, at our institution, emergent surgery has been required for post-EVAR BI in two patients with prior colorectal resection (data not shown). Further research on the correlation between left-sided colectomy and AAA repair is needed.

This study has several limitations. First, this report presents a small number of cases based on the clinical experience at a single institution, and there was no control group. Second, the intensity of ICG fluorescence was not quantitatively evaluated. Nevertheless, this ICG fluorescence imaging could be a simple and reliable option when performing SFC resection in patients with an occluded IMA.

## Conclusions

We performed SFC resection in two patients with an occluded IMA under ICG guidance. To our knowledge, this is the first report on the value of ICG imaging in patients with an occluded IMA who require CRC surgery. We discussed in this report that we should not underestimate the impact of prior colectomy on the risk of BI after AAA repair. In conclusion, we propose that ICG imaging can be used in cancer surgery with altered blood flow to ensure safety, particularly when the patients are vulnerable to extended surgical resection.
